# The Roles of Competition and Mutation in Shaping Antigenic and Genetic Diversity in Influenza

**DOI:** 10.1371/journal.ppat.1003104

**Published:** 2013-01-03

**Authors:** Daniel Zinder, Trevor Bedford, Sunetra Gupta, Mercedes Pascual

**Affiliations:** 1 University of Michigan, Department of Computational Medicine and Bioinformatics, Department of Ecology and Evolutionary Biology, Ann Arbor, Michigan, United States of America; 2 University of Edinburgh, Institute of Evolutionary Biology, Edinburgh, United Kingdom; 3 University of Oxford, Department of Zoology, Oxford, United Kingdom; 4 Howard Hughes Medical Institute, University of Michigan, Ann Arbor, Michigan, United States of America; 5 Santa Fe Institute, Santa Fe, New Mexico, United States of America; Imperial College London, United Kingdom

## Abstract

Influenza A (H3N2) offers a well-studied, yet not fully understood, disease in terms of the interactions between pathogen population dynamics, epidemiology and genetics. A major open question is why the virus population is globally dominated by a single and very recently diverged (2–8 years) lineage. Classically, this has been modeled by limiting the generation of new successful antigenic variants, such that only a small subset of progeny acquire the necessary mutations to evade host immunity. An alternative approach was recently suggested by Recker et al. in which a limited number of antigenic variants are continuously generated, but most of these are suppressed by pre-existing host population immunity. Here we develop a framework spanning the regimes described above to explore the impact of rates of mutation and levels of competition on phylodynamic patterns. We find that the evolutionary dynamics of the subtype H3N2 influenza is most easily generated within this framework when it is mutation limited as well as being under strong immune selection at a number of epitope regions of limited diversity.

## Author Summary

Influenza A (H3N2) has circulated in the human population since 1968 causing considerable annual morbidity and mortality worldwide. Despite the rapid evolution of the hemagglutinin (HA) protein and strong diversifying selection, the global virus population is characterized by a low standing diversity, evident in the serial replacement of antigenic types and in the ‘cactus-like’ structure of its genealogical tree. Elucidating the mechanisms behind these puzzling patterns is key to understanding the evolution of seasonal (H3N2) influenza. One recent epidemiological model proposes a restricted set of antigenic types whose waves of dominance result from frequency-dependent immune selection. Here we develop a model of limited antigenic diversity that explicitly incorporates mutational processes, and use it to address, first, whether this type of antigenic space is capable of generating the characteristic phylogeny of HA sequences, and second, whether the dynamics of (H3N2) influenza are primarily limited by the arrival of mutations or by the opening of antigenic niches. We conclude that a limited antigenic space can explain the observed phylogenetic patterns and that a limited mutation rate is a key property underlying the dynamics of (H3N2) influenza. Our study provides a general framework for assessing the relative roles of selection and mutation in a variety of infectious disease systems.

## Introduction

Influenza viruses are classified into types A–C, among which influenza A is the most pathogenic. These viruses cause between a quarter to half a million deaths worldwide [1] and tens of thousands of deaths in the US during annual epidemics [2]. The economic burden of seasonal influenza in the US is estimated at more than ten billion dollars in healthcare costs alone [3].

**Figure 1:**
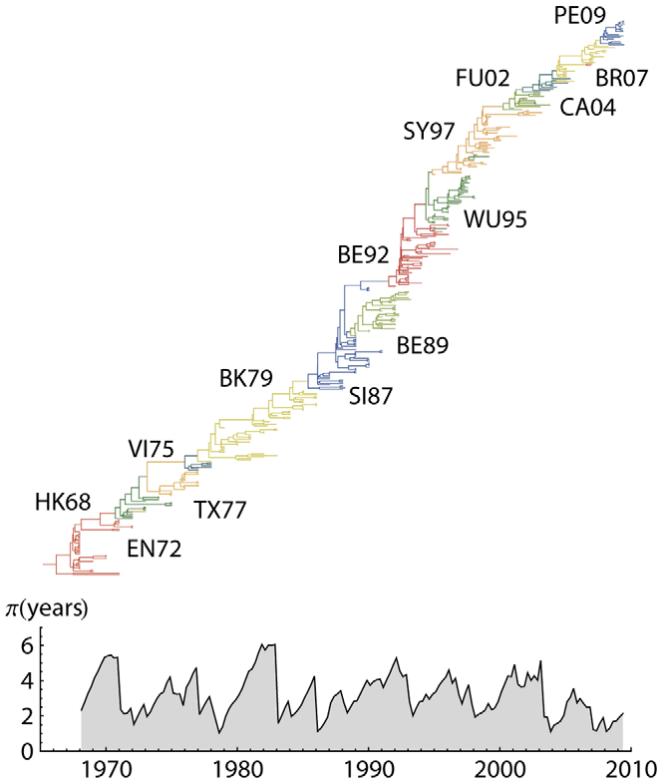
Phylogenetic tree reconstruction of H3 depicting major antigenic clusters and including the associated pairwise diversity. Phylogenetic tree with highest posterior likelihood was reconstructed using 377 representative sequences sampled between 1968–2009. Colors represent estimated antigenic clusters (Hong-Kong 1968 – Perth 2009). Approximately half of the samples include an established cluster annotation [18] and three additional clusters relating to: California 2004, Brisbane 2007, and Perth 2009. Additional sequences were sampled uniformly overtime on a bi-annual scale. Phylogenetic tree was reconstructed using Bayesian MCMC analysis [58], [59] and includes state reconstruction for unannotated sequences and ancestral sequences. Diversity skyline was calculated for the same representative tree. Branches with colors differing from their main neighboring cluster represent uncertainty in the reconstruction, rather than actual cluster changes.

The major targets of humoral immunity against influenza A are its envelope glycoproteins, hemagglutinin (HA) and neuraminidase (NA); these form the basis of its crude classification into subtypes H1N1, H2N2 and H3N2 etc. Since its emergence in 1968, influenza A (H3N2) has continually circulated in the human population. The phylogeny of its HA protein (Figure 1) shows a distinctive ‘cactus-like’ shape with a narrow, usually single-trunked, tree [4], . The ‘narrowness’ of the tree is derived from the fact that contemporaneous H3 proteins share a single common ancestor 2–8 years in the past [6], [7]. This short time is unique to H3N2 given its global spread and its high prevalence and incidence [7].

The classical view of influenza evolution is one of antigenic drift [8], [9], [10] in which antigenic change continually and gradually accumulates in the virus through the influence of selection by way of changes to the HA and NA proteins. By itself, the ‘cactus-like’ structure of the A/H3N2 phylogenetic tree suggests the presence of adaptive evolution [7] and several studies have provided evidence for positive selection [5], [11], [12], [13], [14]. However, it is difficult to explain the limited standing diversity of influenza [15], [16], and the empirical evidence for discontinuous antigenic change [17], [18], under a general antigenic drift framework. Multiple epidemiological hypotheses have been advanced to reconcile the these observations with a process of continual antigenic divergence including short-lived strain-transcending immunity [15], [16], [19], epochal or punctuated evolution [13], [17], trait-space reduction [20] and canalized evolution [21].

A competing hypothesis advanced by Recker et al. [22] eschews the paradigm of antigenic drift, instead considering that, owing to functional constraints on the defining epitopes, the virus population is limited phenotypically to a restricted set of antigenic types. Antigenic types replace each other with waves of dominance resulting from frequency-dependent immune mediated selection as “niches” in antigenic space are dynamically generated and are exploited by the existing virus population. In its original implementation, this model assumes that all antigenic types remain present in the population in low frequencies, as an approximation to the idea that they can be generated by mutation from preexisting strains at a sufficient rate as not to limit the emergence of a type favored by selection. Thus, the model describes in practice a case where influenza outbreaks are caused by host immune selection in a manner that is not limited by the rate of antigenic mutations. Although patterns of turnover are consistent with those observed for H3N2, it is not clear whether the characteristic phylogenetic trees can be generated by this model.

Here, we have attempted to resolve this question using a large-scale individual-based simulation of epidemiological and evolutionary dynamics that allows the complete phylogenetic tracking of a virus population characterized by defined repertoires of polymorphic epitopes. Our model is based on the multi-locus structure employed by [22] with host immunity operating at an epitope-specific level. When contacted by a virus, a host's risk of infection is determined by the number of alleles/epitopes recognized by its immune system. We also introduce the possibility of a long-lived strain-transcending component to the model. Thus, competition between strains is determined both by the number of shared epitopes and a variable level of generalized immunity. Our model differs in this regard from that of Recker et al. [22] which does not permit full cross-protection except in the case of having experienced the exact same combination of epitopes, a feature that implicitly accounts for the effect of highly variable epitopes unique to each strain.

This model structure allows us to make inferences about the roles of mutation and competition in a more general context. Models of antigenic dynamics tend to polarize between those in which the availability of antigenic types dictate the dynamics [13], [17], [23], [24], and those where host immune-mediated selection is the only driver [25], [26]. We refer to the latter regime, where antigenic change is constrained by host population immunity, as selection limited, whereas the former, in which the availability of antigenic mutations pose the rate limiting step, is described as mutation limited. The approach we take in this paper offers a tool for locating influenza on this continuum and would easily generalize to other antigenically diverse infectious diseases.

## Results

**Figure 2:**
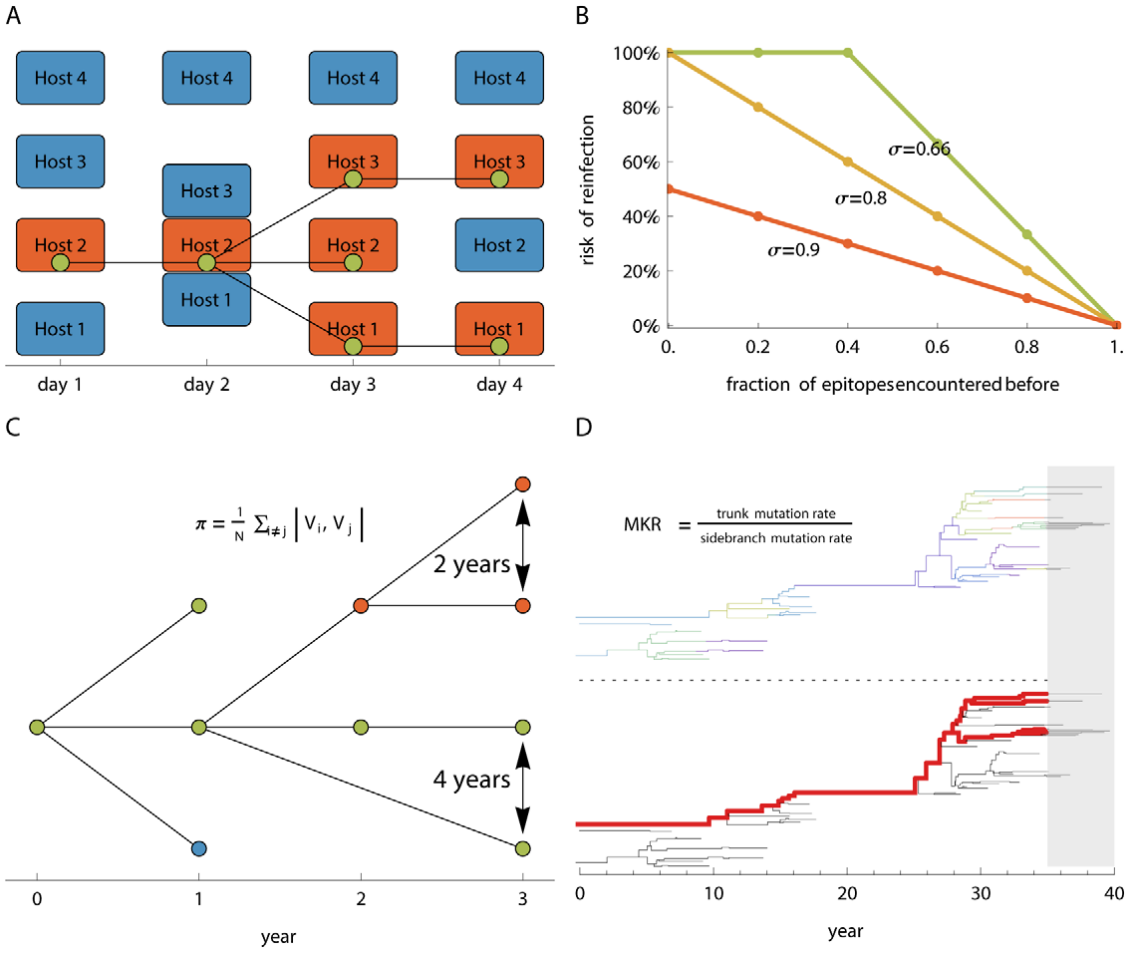
Methods. (**A**) Virus genealogy is tracked at the inter-host level. The genealogy is periodically sampled and the resulting tree is used for analysis. **(B)** Hosts acquire immunity to viral epitopes following infection. Fully naïve hosts are always infected following contact at rate β. The risk of reinfection is based on the similarity to previously encountered strains, as measured through the number of previously encountered epitopes: 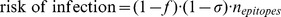 and is at most 100%. Where *f* is the fraction of previously encountered epitopes and σ is the strength of crossimmunity. Lower *σ* values correspond to weaker competition between strains. A form of generalized immunity is attained for σ>0.8 in the five epitope case, relating to a reduced risk of reinfection following previous exposure to any strain. **(C)** Mean pairwise genealogical diversity π is measured by averaging the pairwise distance in years between random contemporaneous samples on the genealogical tree. **(D)** The MK related index is calculated as the ratio of the antigenic mutation rate on the trunk of the genealogy (red) versus the antigenic mutation rate on the sidebranches (black). The trunk of the genealogy was determined by tracing back viral lineages that survived until the end of the simulation and excluding the last 5 years. Antigenic changes are represented by color changes on tree branches (top-tree). The rate of antigenic change on the sidebranches is calculated as the number of antigenic changes on the sidebranches divided by the total length of the side branches in years. The rate of antigenic change on the trunk is calculated as the number of antigenic changes on the trunk divided by the total length of the trunk in years.

We use an individual-based SIR model, explicitly tracking the chains of infection of viral lineages as well as the antigenic phenotype of every virus in the population (Figure 2A). Thus, our model explicitly tracks viral genealogy rather than conducting phylogenetic inference, and therefore does not include any genotype to phenotype map.

A host population of constant size N is simulated with an equal birth and death rate of μ. Infected hosts randomly contact other hosts in the population at rate β. The risk of infection is based on crossimmunity interactions based on their previous unordered infection history [27], [28]. Following infection with a virus displaying a specific antigenic phenotype, hosts acquire partial protection against reinfection with viruses sharing epitopes, and full protection against the exact same strain. There is no super-infection in the model; while infected, hosts are protected from co-infection with other strains. Hosts recover from infection at a constant rate ν. (see Methods for details of the model).

### Antigenic-Phenotype Replacement and Single Phenotype Dominance in an Evolution-Free Framework

**Figure 3:**
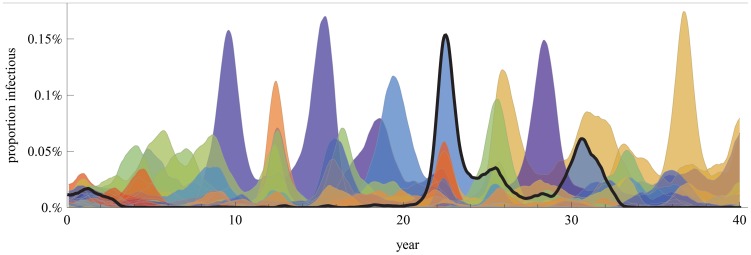
Changes in the proportions of hosts that are infectious with different strains within a 2 variants per epitope, 5 epitope system in an “evolutionary free” framework: for all of the possible 32 strains the existence of at least one carrier was assured and no antigenic mutations were introduced. The superimposed time series were smoothed and ordered back to front by peak prevalence, maintaining the least prevalent strain in the front. The 3th highest peaking strain was outlined as an example. Single strain dominance was calculated based on the quantity ε from [22]. Major peaks of incidence are generally associated with one or two dominant antigenic-phenotypes with ε = 0.36±0.07 (mean ± standard-deviation across 5 simulations) and a myriad of lower prevalence ones. Antigenic-phenotypes reemerge with alternating frequency. This simulation includes a single homogeneously mixed host population of 40M hosts, contact rate β = 0.6 and a 4 day recovery rate. Each epitope unencountered by the host contributes to a 17.5% increase in the risk of infection with a different strain (see methods for full description of epidemiological parameters).

To explore the epidemiological dynamics of our model in isolation, we implemented a parameterization lacking mutation, in which extinction was preempted by maintaining at least one carrier for each antigenic-phenotype. Different colors (Figure 3) represent the prevalence of different antigenic-phenotypes. Here, the antigenic repertoire is derived from combinations of variants at 5 distinct epitopes (see Methods for full description of epidemiological parameters).

A mutation-free model can result in different alternative dynamics based on model parameters ranging from stable coexistence of completely discordant antigenic-phenotypes to the successive replacement of strains through chaotic or cyclic behavior [25]. Not surprisingly, our model implementation with no explicit evolution also generates these waves of replacement (Figure 3), suggestive of H3N2 influenza as proposed by Recker et al. [22].

### Phylodynamic Patterns across Different Mutation Rates

We examined the effects of mutation at different rates on the resulting phylodynamic patterns of the virus by seeding the population with a single strain and tracking antigenic and evolutionary changes. We measure diversity π as the average time separating two randomly selected contemporaneous viruses since their divergence from a common ancestor. Because branch lengths in our genealogies are measured in years, the resulting diversity is also measured in years.

**Figure 4:**
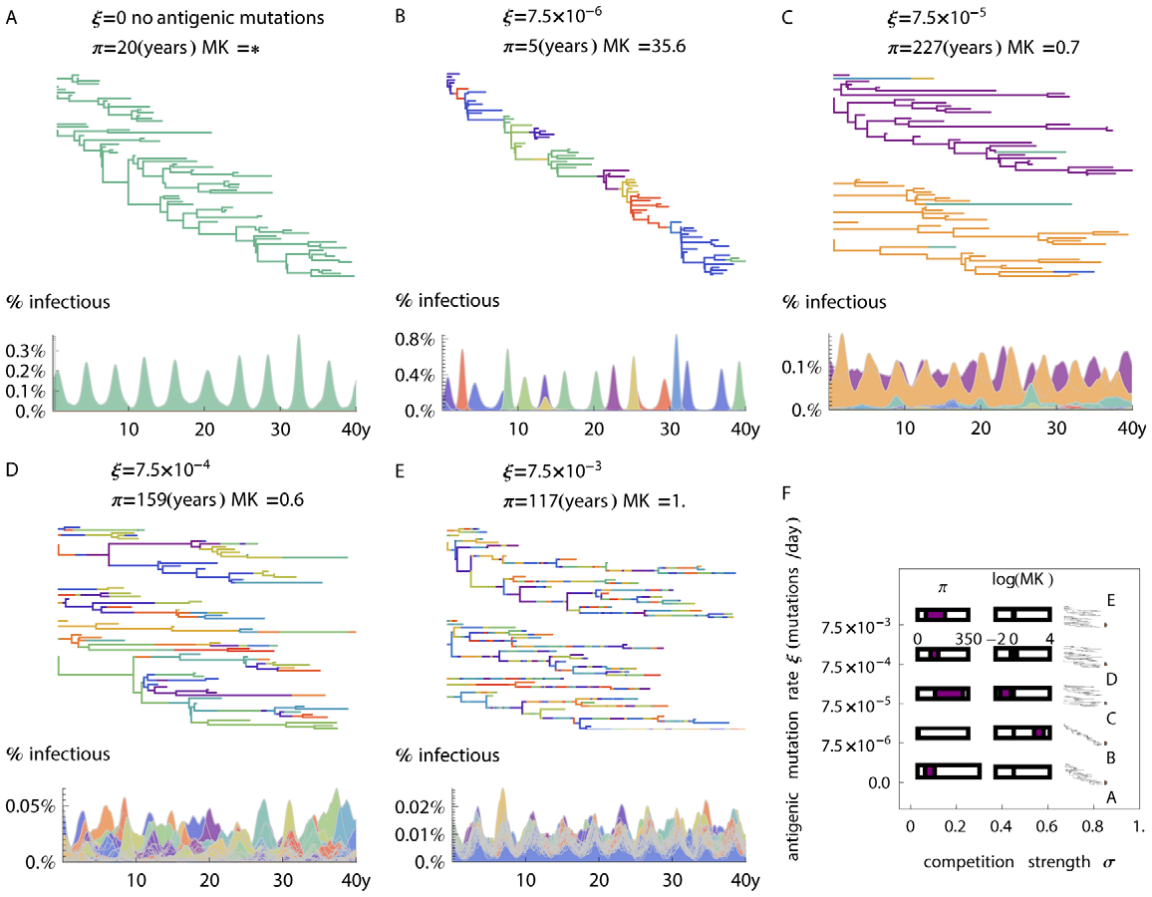
Changes in the proportions of hosts that are infectious with different strains and the related phylogenetic behavior with increasing mutation rate. Phylogenetic trees are based on samples of directly measured virus genealogy in the simulation, and only the last 40 years are visualized in the figure (for the complete genealogy over the whole time period see ). Diversity π is calculated as the mean distance, measured in years, for the coalescence of random pairs of contemporaneous samples in a tree. The MK related index (MKR) is calculated as the ratio of the antigenic mutation rate on the trunk (fixed) versus the antigenic mutation rate on the sidebranches. **(A)** Model with no mutation, a single antigenic type persists under neutral evolution. When there are no antigenic mutations a genealogical tree which follows neutral viral evolution exists. Genetic diversity for this tree relates to population dynamics only – to incidence and the prevalence. **(B)** Model with low mutation rate of ξ = 7.5×10^−6^ antigenic-mutations per day. Successive strain replacement with higher epidemic peaks is observed. Rare antigenic mutations are advantageous and are more likely to fix and have viable offsprings, consequently lowering genetic diversity. **(C)** The introduction of a higher mutation rate ξ = 7.5×10^−5^ leads to antigenic and genetic divergence. Dynamics are ruled by endemic or cyclic behavior of discordant antigenic strains. Mutations are more likely to be deleterious, facing competition from the two prevalent strains. Phylogenetic patterns include two deep branches representing each strain and a low rate of coalescence between strains. **(D)** For a mutation rate ξ = 7.5×10^−4^ epidemiological behavior resembles the evolution free framework (Figure 3). Phylogenetic patterns exhibit high genetic diversity and weak negative selection pressure. **(E)** Loss of strain structure due to high mutation rate ξ = 7.5×10^−3^. At this high mutation rate the antigenic traits are no longer heritable and each linage displays a constantly varying antigenic phenotype. No selection forces are measured and genetic diversity is expected to be determined by random coalescence. **(F)** Summary statistics and typical trees for varying mutation rates and fixed crossimmunity (filled area within rectangles indicates 1σ confidence interval for 5 repeated runs). Simulation parameters are the same as those described in Figure 3, but include the possible extinction of strains, and mutations to individual epitopes at a specified rate ξ (see methods for full description of epidemiological parameters).

In the absence of antigenic mutation, only a single strain persists, experiencing transient oscillatory dynamics between near extinction, and endemic equilibrium conditions (Figure 4A). As all viral traits are equal, there are no selective forces and the observed phylogeny and coalescence rates can be directly related to prevalence and incidence [29]. This yields random coalescence within contemporaneous viral lineages and a large associated pairwise genetic diversity (π = 30±12 years; mean±std across 5 simulations).

For low mutation rates (Figure 4B) the introduction of new mutations is the critical determinant of strain dynamics. Each new variant outcompetes the one that came before, resulting in a spindly phylogenetic tree and therefore low diversity (π = 5.7±0.8 years). Temporally adjacent strains are antigenically similar, rather than discordant, forcing strong competitive exclusion and single strain dominance (ε = 0.93±0.02), where ε is the proportion of infections caused by the most common strain.

An increase in mutation rate (Figure 4C) leads to deeper branches with a corresponding increase in phylogenetic diversity (π = 220±100 years) and more pronounced antigenic divergence. Here, the population dynamics are ruled by the endemic or cyclic behavior of discordant antigenic sets. The emergence of new intermediate antigenic types is suppressed by competition from the two prevalent strains [25].

At a relatively high mutation rate (Figure 4D), we approach population dynamics similar in appearance to those of the mutation-free model (Figure 3). Diversity is high (π = 120±30 years), with deep yet occasionally coalescing branches.

In general, a threshold exists at which mutation overwhelms selection resulting in a population drifting away from the fittest genotype [30]. For sufficiently high rates of antigenic mutation, all antigenic types reach near equal frequency in the population (Figure 4E). On a population scale, the high mutation rate weakens frequency-dependent selection and results in the breakage of antigenic strain structure; antigenic types do not cluster across the genealogy. The loss of selection forces breaks down phylogenetic structure and leads to a reduction in the depth of the branches (π = 120±70 years) compared to the one observed for discordant antigenic types.

### Measuring Selection Strength and Direction

By comparing fixation versus extinction of antigenic mutations, using a quantity related to the McDonald-Kreitman (MK) index [31], [32], [33] we estimated the strength and direction of selection on antigenic mutations in our model (see Methods). Here we calculate an MK related (MKR) index as the ratio of the per-year rate of antigenic mutation on the trunk to the per-year rate of antigenic mutation on the side branches.

If antigenic mutations are advantageous for long-term virus persistence, an MKR ratio above 1 is expected. In this case, individuals exhibiting these antigenic mutations will be more likely to fix in the population and contribute to substitutions on the trunk of the phylogeny. Similarly, if antigenic mutations are deleterious to the long-term success of the virus, an MKR index of less than 1 is expected. This is because mutant individuals will tend to be lost from the population and side branches will show an excess rate of substitution.

We find that, when rare, antigenic mutations show highly increased rates of fixation (MKR = 19±11), and therefore evidence of strong positive selection (Figure 4B). Hence, we find that strong positive selection results in both a spindly tree and an overabundance of antigenic mutations of the trunk of the phylogeny. An increase in the mutation rate leads to the emergence of antigenically discordant types, and the suppression of other antigenic mutants (Figure 4C); here, we find strong negative selection mediated by host immunity with an MKR index of 0.47±0.22. At a still higher mutation rate (Figure 4D), we observe a balance of positive and negative selection resulting in MKR = 1.1±0.3. With saturating mutation rates (Figure 4E) we further lose the signature of selection (MKR = 1.0±0.1) on phylodynamic patterns.

### Relationship between Cross-immunity, Mutation and Selection

Our model contains a cross-immunity parameter σ which allows us to explore a range of immune selection regimes: when σ = 1, we have full cross-protection (as might arise if each epitope elicited a very strong immune response) and when σ = 0, cross-protection between strains is only high if they share their entire variable repertoire.

**Figure 5:**
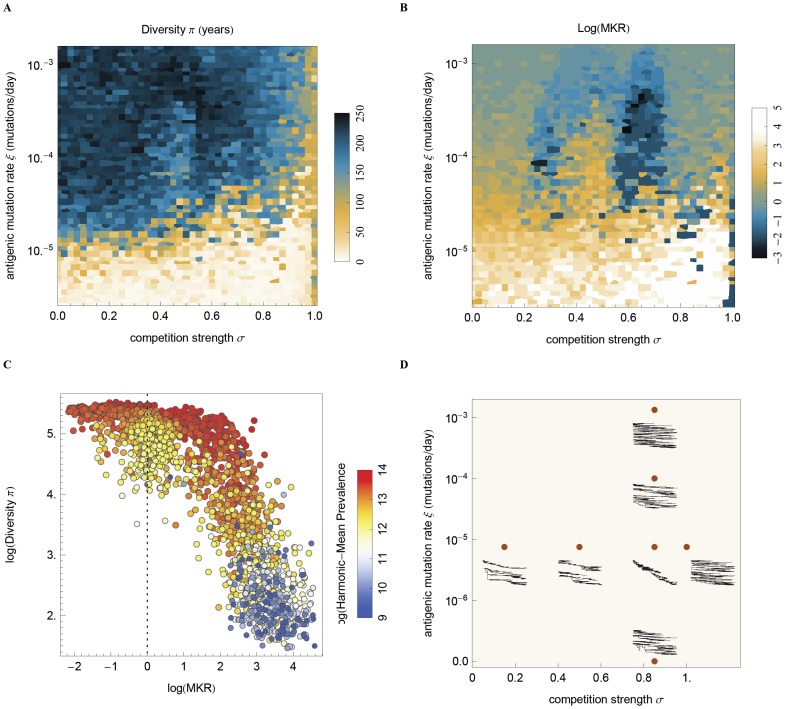
Changes in genetic diversity and the McDonald-Kreitman related index (MKR) for varying strengths of strain competition and antigenic mutation rates. **(A)** Mean pairwise genetic diversity π is measured as the mean distance, measured in years, for the coalescence of random pairs of contemporaneous samples in a tree. Diversity measurement is capped by twice the simulation run length which amounts to 240 years. **(B)** The MKR is measured as the ratio between the trunk antigenic mutation rate (fixed) and the sidebranches antigenic mutation rate. Evidence of positive selection is observed when the MKR index is significantly above one, and negative selection when it is significantly below one. Areas of strong positive selection are associated with lower genetic diversity as a small subset of the population contributes to long term viral evolution. Strong negative selection is associated with disruptive selection maintained by existing strains. **(C)** Diversity for varying strengths and directions of selection as measured by the MKR index. Diversity decreases with stronger positive selection *ρ* = −0.85 (Pearson's correlation right of dotted line), and increases for stronger negative selection *ρ* = −0.28. The harmonic mean of the prevalence is also strongly correlated with genetic diversity *ρ* = 0.76 (heat map). **(D)** Typical trees for varying strengths of strain competition and antigenic mutation rate. Effective competition combined with a limited availability of antigenic mutations results in narrower trees with lower pairwise diversity. This figure was parameterized to use R0 = 3 and a population size of 50M to limit stochastic extinctions for a large parameter range (see methods for full description of epidemiological parameters).

In general, stronger cross-immunity results in lower prevalence as hosts fail to be re-infected (Figure S1). We find that, for most of the parameter space, genetic (genealogical) diversity π, increases with weaker cross-immunity and with more rapid mutation (Figure 5A). The (mostly) monotonic relationship between competition and diversity is broken at the threshold of limiting similarity [34] where, regardless of epitope differences, two strains suffer full cross-protection. This scenario, shown as a band on the right-hand side of Figure 5A where σ = 1, results in the disappearance of selective effects and greater levels of genetic diversity. Here, diversity rebounds to its neutral expectation due to random coalescence. Exceptions to the monotonic pattern of diversity with competition can also be found for intermediate mutation rates.

The relationship between mutation, cross-immunity, and the MKR index is less straight-forward (Figure 5B). Here, the highest levels of positive selection are present when cross-immunity is strong (σ = 0.8–0.9), and mutation is weak (ξ = 10^−5^). When mutation rate is limiting (ξ<5×10^−5^) then antigenic mutations are favored by natural selection (MKR>1). However, when mutation rates are higher (5×10^−5^<ξ<10^−3^), negative selection by-and-large predominates. The strongest negative selection occurs in a region of moderate cross-immunity (σ = 0.6) corresponding to previously observed discordant dynamics (Figure 4C).

There is also a clear relationship between diversity and selection as measured by the MKR index (Figure 5C). We observe a strong negative correlation between MKR and levels of diversity (*ρ* = −0.86; Pearson's correlation). If we separate results into a regime of positive selection (MKR>1) and a regime of negative selection (MKR<1), we observe similar results within each regime. Stronger positive selection coincides with a decrease in genetic diversity (*ρ* = −0.85 when MKR>1), and stronger negative selection tends further increase diversity through the persistence of discordant strains and associated deep branches (*ρ* = −0.28 when MKR<1). As expected from population genetic theory [29], increases in viral prevalence also coincide with increases in viral diversity, however, the correlation is weaker under positive selection (*ρ* = 0.74 when MKR>1) than under negative selection (*ρ* = 0.87 when MKR<1) and cannot be trivially dissociated from the effects of selection.

Two additional strain diversity measurements based on the ecological dynamics are shown in Figure S2, the Shannon diversity index and the level of single strain dominance (Methods). Similar to genetic diversity, positive selection is correlated with an increase in single strain dominance (*ρ* = 0.88 when MKR>1) and a decrease in Shannon diversity (*ρ* = −0.88 when MKR>1). Negative selection decreases Shannon diversity (*ρ* = 0.39 when MKR<1) and increases single strain dominance (*ρ* = −0.23 when MKR<1). While negative selection lowers the number of circulating strains, it increases genetic diversity π through the existence of deep non coalescing branches.

### H3N2-like Characteristics

The patterns described so far suggest that the dynamics of H3N2 influenza within this framework correspond to a regime in which host immune mediated selection is strong and the antigenic mutation rate is low. We now extend the model in order to examine other characteristics relevant to H3N2 in a more detailed epidemiological setting that includes seasonality and a basic global population structure.

In this analysis we include three demes representing the northern hemisphere, the southern hemisphere and the tropics. Northern and southern hemisphere demes experience an opposing seasonal modulation (with a 14% amplitude and six months phase difference) while tropical regions experience two weaker seasons annually [35] (see Figure S3 and Methods). In addition the southern hemisphere population is reduced in comparison to northern hemisphere and tropical populations (Methods).

**Figure 6:**
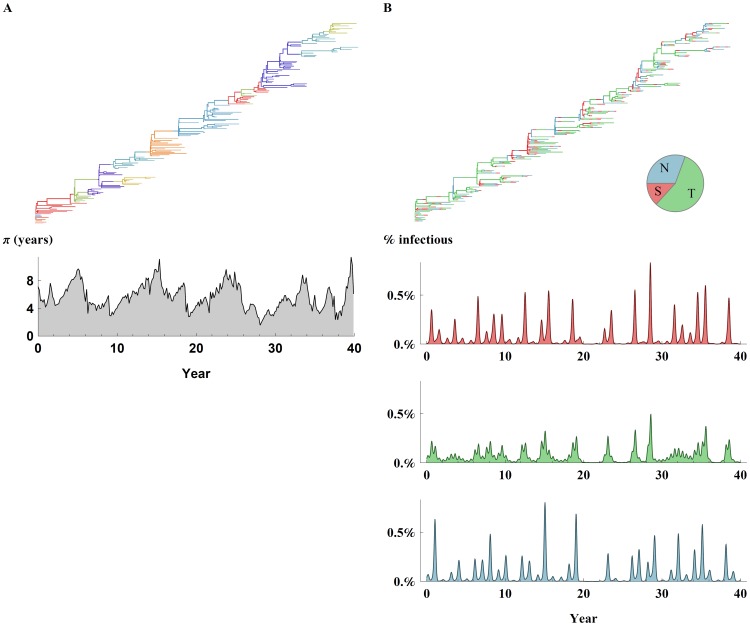
H3N2 like characteristics. Using a model with an alternative epitope configuration and three global demes representing the northern hemisphere N, southern hemisphere S, and the tropics T. **(A)** Top - typical tree colored by antigenic clusters (see Methods). On average 13±6 clusters lasting 4±2 years come to dominate the virus population over the course of the 40 year simulation. Bottom - Genealogical diversity (π) displaying “boom & bust” patterns (10%–90% range of 3–9.5 years) associated with H3 diversity with an average of 5.7±0.1 years. **(B)** Top - typical tree colored by deme. The tropics metapopulation has a higher proportion in establishing the trunk (68%±9) of the phylogeny followed by the northern (20%±8) and the southern (12%±3) population. Bottom – proportion of hosts infected in the northern hemisphere, tropics and southern hemisphere. Epitope configuration for this figure was 5×4×3×2 variants per epitope, 4 epitope system. Average yearly incidence in the northern and southern hemisphere demes is (5.7%±0.1, 5.8%±0.1) respectively, while incidence in the tropics is slightly lower 5.5%±0.1. Annual epidemics are generated almost regularly yet display a high level of variability in peak size in the northern hemisphere (CoV = 1.1±0.1, coefficient of variation) and lower variability in the tropics (CoV = 0.7±0.1). In this simulation 42M hosts were divided to three demes with the tropics and the south having 16M hosts and the southern hemisphere having a lower 10M population. Annual seasonal patterns were established for the temperate demes, and biannual weaker seasonality in the tropics (see methods for full description of epidemiological parameters).

In this model we use an antigenic repertoire with 4 epitopes differing in the number of alternative variants per epitope. A typical tree for this configuration together with the corresponding diversity skyline is depicted in Figure 6A. We observe 13±6 antigenic clusters that come to dominate the virus population over the course of the 40 year simulation (Figure S4-A) with an average duration of 4±2 years. Clusters are defined based on cumulative changes in two or more epitopes based on [36] (Methods). The turnover of virus strains results in a characteristic spindly phylogenetic tree and low standing genetic diversity (π = 5.7±0.1 years). Over the course of the 40-year timespan, genetic diversity experiences a boom and bust pattern (Figure 6A) with a 10%–90% range of 3–9.5 years measured by combining diversity skylines of five repeated simulations. The repeat of exact antigenic types is uncommon in the model (Figure S5) while epitopes with more restricted variability (2–3 variants) frequently reemerge (Figure S6).

Average yearly incidence in the northern and southern hemisphere demes is (5.7%±0.1, 5.8%±0.1) respectively (Figure 6B), while incidence in the tropics is slightly lower 5.5%±0.1. Annual epidemics are generated almost regularly yet display a high level of variability in peak size in the northern hemisphere (CoV = 1.1±0.1, coefficient of variation) and lower variability in the tropics (CoV = 0.7±0.1). The interquartile range in peak weekly cases ranges (IQR≈200–800 cases per 100000) in the northern hemisphere and (IQR≈400–1100) in the tropics.

In this model the tropics or lower and mixed seasonality populations exhibit a greater role (68%±9) in establishing the trunk of the influenza tree (Figure 6B). The southern hemisphere experiences a smaller (12%±3) part in establishing the trunk of the tree in comparison to the northern hemisphere (20%±8). In addition we find that antigenic variants are more likely to reach significant prevalence in the tropics earlier making the tropics “antigenically ahead” (Figure S4-B). Antigenic variants reach 5% of their total deme prevalence 2±1.5 months earlier in the tropics compared to the northern hemisphere and 3±2 months earlier in the tropics compared to the southern hemisphere (p<0.001 for the combined results). Similarly antigenic variants decline (reach 95% of their total prevalence) earlier (1.7±0.3 month, p<0.0005) in the tropics compared to the northern hemisphere, yet not significantly earlier or later than the southern hemisphere.

We find that strong competition and high R0 values generates more regular annual epidemic peaks while maintaining low genetic diversity. In addition we find that within antigenic cluster evolution also contributes to maintain low genetic diversity. An increase (0.005 compared to 0.001 of contacts) in the strength of the metapopulation coupling slightly improves the epidemiology by decreasing the likelihood of long periods without annual epidemics.

To establish whether low genetic diversity can be maintained when the number of epitopes or variants per epitope is increased we repeated the same parameterization with double the number of epitopes and with twice the number of variants per epitope and by keeping either the per-epitope or overall mutation rate. The results are summarized in Table S1. When doubling the number of epitopes, the model can attain similar results with respect to genetic diversity π and overall incidence, when the total mutation rate across epitopes is maintained and the cross-immunity decrease per–epitope change is halved. In contrast, it is not clear whether a model that includes an increase in the number of variants per epitope can maintain low genetic diversity levels and maintain similar or higher incidence levels.

## Discussion

Herein, we implemented an individual-based model that allowed us to track both the ecological and evolutionary dynamics of a pathogen population, in which cross-immunity is orchestrated by a finite set of antigenic loci of limited variability [22]. We used this model to compare phylodynamic patterns under a regime governed primarily by limitation on the introduction of antigenic mutations (mutation limited), to a regime determined by the availability of antigenic niches (selection limited), and under varying strengths of competition between strains. We use this framework to determine the conditions under which a limited antigenic repertoire could explain the observed phylodynamic patterns of H3N2 influenza.

Explicit modeling of evolution, through the introduction of antigenic mutation at different rates, allows us to consider phylogenetic trees in addition to epidemiological dynamics. Resulting phylodynamic patterns range from successive strain turnover, to discordant antigenic sets, to dynamics resembling those of a model lacking explicit evolution and finally to the collapse of antigenic structure. Each of these can be explained by the interplay of selection and mutation, as measured here through the MKR index, and by considering different strengths of immunity generating competition between strains.

The dynamics of our individual-based model are generally in good agreement with the epidemic behavior of influenza A (Figure 6). Like observed epidemiological patterns [17], [23], [37], [38], [39], [40], [41], annual temperate climate epidemics occur almost regularly with substantial year-to-year variation in incidence (CoV = 1.1±0.1 compared to (CoV = 1.0±0.2) in literature survey. Observed temperate climate annual attack rates of influenza A (H3N2) are slightly higher, approximately 8% from 1976 to 1981 [38] compared to 5.8%±0.1 in simulation, while peak epidemic weakly cases are higher in the simulation (IQR≈200–800 cases per 100000) in comparison to (IQR≈130–380, IQR≈80–240) in [37] and [40] respectively. The tropics exhibit lower and weaker seasonality (Figure 6, Figure S3) with slightly lower yearly attack rates (5.5%±0.1) and substantially lower prevalence (Figure 6). In agreement with antigenic cartography [18], [42] 13±6 clusters dominate the global world population (Figure S4-A) with an average duration of 4±2 years, exhibiting mostly the dominance of 1–2 clusters globally. With respect to individual epitope changes we find the model reproduces the observation of the tropics being “antigenically ahead” [23], giving rise to antigenic changes 2.5±1.5 month ahead of the northern and southern hemisphere (Figure S4-B) and showing decline in antigenic variants 1.7±0.2 month earlier than the northern hemisphere. In agreement with observed phylodynamic patterns [43] the tropics metapopulation has a higher proportion in establishing the trunk (68%±9) of the phylogeny followed by the northern (20%±8) and the southern (12%±3) population. The higher contribution of East and South-East Asia as the origin of H3N2 globally circulating lineages is hypothesized to originate from lower and mixed seasonality in these regions and is consistent with our model [23]. The key difference between the hemispheres in the model being, lower population size in the southern hemisphere with proportionally lower contact rate between the meta-populations.

Refinement of the epidemiological model, such as the inclusion of an exposed period, can further improve the comparison to empirical data. In particular, the above properties were obtained with a basic reproduction number of R0≈3.24, on the upper bounds of current estimates for seasonal influenza. This value can possibly be decreased by considering such an extension.

We find that a model with 4 epitopes and a low but variable number of variants per site, an antigenic mutation rate of ≈10^−5^ per day and a reduction of cross-immunity of 13% per epitope results in phylodynamic patterns broadly consistent with those seen in H3N2 influenza (Figure 6). When doubling the number of epitopes, the model maintained similar results with respect to genetic diversity π and overall incidence, when the total mutation rate across epitopes was maintained and the cross-immunity was modified to a 6.5% per epitope change. These parameters are quite comparable to parameters used in other models of influenza evolution. For example, Koelle et al. [18] use 5 epitopes with mutations of either large or small antigenic effect. Small mutations reduce cross-immunity by 7% and occur at a rate of ∼5×10^−4^ per day, while large mutations reduce cross-immunity by 20% and occur at a rate of ∼10^−5^ per day. In the model of Bedford et al. [21] mutations reduce cross-immunity by between 1% and 11% (95% bounds), but occur at a faster rate of 10^−4^ per day. Ferguson et al. [17] find that a model with 12 codons, each with 20 amino acid variants, in which mutations occur at a rate of 3×10^−5^ per day and reduce cross-immunity by ∼7% gives restricted diversity without short-term strain-transcending immunity, and 1.2×10^−4^ per day, when transient immunity is included. From this, it seems clear that models involving a slow influx of antigenic mutants of around 10^−5^ per day are generally capable of producing influenza-like patterns of restricted diversity.

Increasing host population size in the model results in an increase in viral genetic diversity, as more opportunities for antigenic mutation arise within the larger host population. Thus, scaling competitive interactions between strains, and/or antigenic mutation rate, is required to maintain limitations on the effective exploration of antigenic space. In addition, other epidemiological phenomena, besides low antigenic mutation rates, may also contribute to limit the rate at which novel antigenic phenotypes emerge in the influenza population. These may be provided by population structure and the seasonality of transmission [23], [44], as well as by short-term strain-transcending immunity, which was found capable of limiting genetic and antigenic diversity in a similar model with a much larger antigenic space [16] and in a limited diversity antigenic model [45]. However, a global metapopulation structure is not expected to be the dominant cause behind the low standing genetic diversity of influenza. Influenza B exhibits similar epidemiological dynamics, and lower prevalence, yet it exhibits much higher genetic diversity through the co-circulation of multiple lineages [16], [46]. Also, a more complex metapopulation structure with multiple patches can either increase genetic diversity by facilitating the coexistence of viruses at different weakly coupled patches, or decrease genetic diversity through the generation of population bottlenecks. The role of variation in viral fitness is an important consideration in future studies, particularly in light of recent observations linking binding properties of HA with antigenic escape [47]. The empirical finding of a non-trivial relationship between virus fitness in susceptible individuals and immune evasion was suggested as a possible alternative mechanism for generating positive selection pressure on antigenic sites and for limiting antigenic diversity [47].

Future work should investigate quantitative patterns and statistical approaches for discriminating among the different models and associated hypotheses that currently exist in the literature and for inferring the relative importance of the mechanisms they represent, keeping in mind that the models are not necessarily mutually exclusive. At the same time, empirical advances on the molecular basis of immune evasion and recognition, on the genotype-to-phenotype map, and on epitope identification and population serology, will allow a better evaluation of the models' assumptions, including the representation of serological space.

In common with the Recker et al. [22] model and in contrast with other phylodynamic models [16], [17], [21], we find here that antigenic epitopes are frequently recycled (Figure S6). Importantly, this does not mean that such recycling is observed for the antigenic types (epitope repertoires) themselves, since the same antigenic type only re-emerges at long intervals (Figure S5) and rarely in the course of 40 simulated years. It's possible that such reemergence could explain the antigenic cross-reactivity between sera from around the 1918 H1N1 pandemic and viruses emerging in the 2009 H1N1 pandemic [48], [49], [50], [51]. However, antigenic stasis of the swine lineage leading to the 2009 pandemic could also explain these observations. Much further work on epitope identification and population-wide serological surveys is necessary to establish the validity of this model's prediction on the re-cycling of constituent low diversity epitope variants (Figure S6). Nevertheless, several empirical observations are becoming available that are consistent with such recycling and the subject is discussed in detail in the companion paper [52]. For example, an antigenic analyses performed on H2N2 influenza, a number of monoclonal antibodies raised against a 1957 strain were shown to cross-react strongly with a strain isolated in 1964, yet not with the 1963 strain [53]. In Reichert et al. [54] the hemagglutinin of both novel pandemic H1N1 and pre-1940 H1N1 lack specific glycosylation sites on the globular head of HA1. These reverse glycosylation patterns were suggested to possibly shield antigenic sites for a timescale of decades and in so doing, to effectively contribute to their recycling and to the age distribution of cases. In Bui et al. [55], several protective antigenic and T cell H3 epitopes show temporal variability across drift variants, with two of these specifically exhibiting a decrease and increase in conservancy consistent with epitope “recycling”. Post translational and conformational changes may hinder the validity of this analysis especially for epitope 1 which acquired two surrounding glycosylation sites. In Wang et al. [56], mice Anti-H3 mAbs were shown to neutralize H3 viruses that span 40 years, as measured by immunofluorescence against MDCK cells (see Table 2 in [56]). All three mAbs (see Figure 4 in [56]) displayed variability in their ability to neutralize H3 viruses for lower concentrations (<15 µg/ml of 7A7 and <25 µg/ml for the other two) in plaque reduction assays. For example for mAb 7A7 neutralization was better for HK68, than diminished for BJ92 and then increases for PAN99 and BRIS07. This pattern could also be due to secondary effects of amino acid differences outside the actual epitope as well through structural effects, but effectively behaves as epitope recycling over substantial durations of many years.

In conclusion, within our framework, the rate of antigenic mutation was found to strongly influence whether selection was positive or negative, and hence, the topology of the tree and associated diversity of the virus. Strong positive selection is generated by effective competition under low mutation rates, and results in spindly trees with low genetic diversity. In this regime, antigenic mutations often fix in the virus population, lowering genetic diversity, as consistent with H3N2. An increase in mutation rate across a broad spectrum in competition strength, leads to negative selection and generates antigenic divergence. This can potentially result in the coexistence of discordant antigenic types repressing the emergence of antigenic hybrids, through strong negative selection on antigenic change, with each discordant antigenic type maintaining a deep phylogenetic branch. Although not strictly mirroring the assumptions about development of the Recker et al model, our framework strongly implies that limitations on antigenic architecture alone are unlikely to reliably reproduce “skinny' trees and some restrictions on mutation rate and/or other considerations such as fitness differences are likely to play a role. It is important to note that this exercise does not also privilege other hypotheses concerning diversity restriction in influenza as these also are strongly sensitive to mutation rate. Overall, it emphasizes that phylogenetic patterns do not serve as a discriminatory tool between these by no means mutually exclusive hypotheses. However, they can provide a basis to exclude specific hypotheses and offer a means by which the contributions of mutation and selection can be assessed. Needless to say, the latter has important implications for the updating of vaccines against influenza. Under a mutation-limited regime, a hypothetical vaccine should be effective until a new antigenic variant is introduced to the population through migration or mutation. Alternatively, when a limited number of alternative but conserved epitopes are continuously circulating with their abundance determined through competitive interactions and immune mediated selection, a vaccine against one of them may lead to rapid strain replacement, while a vaccine against all of them may result in effective intervention.

## Materials and Methods

### Cross Immunity

We assume that a strain's antigenic attributes are determined by a set of separate epitopes and that each epitope contains a discrete number of alternative variants. Thus, a strain's antigenic properties are defined by an *n*-tuple with *k_i_* variants per epitope giving a possible number of 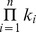 antigenically distinct strains. Hosts acquire immunity to viral epitopes following infection. Fully naïve hosts are always infected following contact at rate β. The risk of reinfection with the exact same strain is always zero. The chance of reinfection with a different strain is based on the similarity to previously encountered strains, measured through the fraction of previously encountered epitopes: 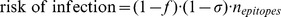 and is at most 100%. Where *f* is the fraction of previously encountered epitopes and σ is the strength of crossimmunity. Lower σ values correspond to weaker competition between strains. A form of generalized immunity is attained for σ>0.8 in the five epitope case, relating to a reduced risk of reinfection following previous exposure to any strain (Figure 2B).

### Mutations

Phenotype changes are driven by mutation events. Mutations change the antigenic properties of a strain but do not influence the shape of the genealogical tree directly; the tree shape will be determined by selection, epidemiological dynamics and the stochastic processes favoring a specific isolate and its offsprings, implicitly rather than explicitly. Mutations involve changes in a single epitope; this makes some phenotypic changes more attainable in comparison to others, even for high mutation rates. The antigenic mutation rate ξ gives the per-day probability for a virus to mutate in a single epitope site.

### Genealogy Tracking and Related Diversity Quantities

The genealogy of the virus population was tracked directly throughout the simulation (Figure 2A). Constant random sampling of viruses was performed periodically. Genealogical pairwise diversity (π) was measured by averaging the time unit distance on the tree between random contemporaneous sample pairs (Figure 2C). This quantity relates to pairwise genetic diversity, as measured on an accurately reconstructed phylogenetic tree. Diversity measures are limited in our simulation to a maximum total of twice the total running time of the simulation which amounts to 240 years. Although it's clear that some parameter ranges would show diversity greater than 240 years, they will saturate at this threshold (Figure 5C).

The MKR index was calculated by dividing the observed rate of occurrence of phenotypic mutations on the trunk of the tree, by the per-year mutation rate on the side branches (Figure 2D). This allows us to estimate the importance of antigenic mutations on the likelihood of fixation of a given viral linage.

Single strain dominance was calculated based on the quantity ε from [22] and is calculated using the following formula:
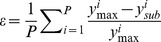
(1)where y_max_ and y_sub_ are the prevalence of the most and 2^nd^ most prevalent strains. The normalized difference between the two is averaged across P epidemic peaks.

### Increasing Mutation Rate Parameters

For figures 3, 4 and S7, R0 was set to ≈2.4. Antigenic diversity was limited to 2 variants per epitope to attain tractable results for a wide range of mutation and cross-immunity parameters, and for the same reason no metapopulation structure was established (Table 1).

**Table 1:** Increasing mutation rate model parameters.

Parameter	Value
population size N	40,000,000
contact rate β	0.6[1/day]
recovery rate ν	0.25[1/day]
birth/death rate μ	1/25[1/year]
Epitopes	5
variants per epitope	2×2×2×2×2
cross-immunity σ	0.825

### Mutation-Competition Simulation Parameters

For this set of simulations (Figure 5) R0 was set to ≈3 and no metapopulation structure was assumed. Higher R0 and longer duration of infection reduce the effect of critical community size over the range of parameters analyzed given the population size determined by computer resource use (Table 2).

**Table 2:** Mutation-competition simulation parameters.

Parameter	Value
population size N	50,000,000
contact rate β	0.6[1/day]
recovery rate ν	0.2[1/day]
birth/death rate μ	1/30[1/year]
epitopes	5
variants per epitope	2×2×2×2×2

### H3N2 Simulation Parameters

This single simulation parameterization is intended to test whether H3N2-like phylogenetic trees can result from a model with a restricted set of antigenic phenotypes using an alternative epitope configuration and a basic metapopulation structure which includes seasonality. The basic reproduction number was set to R0≈3.24. In this case three demes were assumed, representing the northern hemisphere, the southern hemisphere and the tropics. Four epitopes with a variable number of variants per epitope were used. Contact rate was attenuated sinusoidally for southern and northern hemispheres to establish seasonal patterns. Tropical climate seasonality was sinusoidally modulated include two seasons [35] of weaker amplitude [57] (Table 3).

**Table 3:** H3N2 simulation parameters.

Parameter	Value
population size N North/Tropics/South	16M/16M/10M
contact rate β_0_	0.6[1/day]
Between deme contact	0.005[1/day]
recovery rate ν	0.185[1/day]
birth/death rate μ	1/30[1/year]
epitopes	4
variants per epitope	5×4×3×2
Temperate Climate Seasonality North/South	14% Jan/July Six Month Phase
Tropics Seasonality	7% Dec/June
Mutation rate ξ	0.000008[1/day]
cross-immunity σ	0.87

### Antigenic Cluster Transitions

It is not clear how changes in individual epitopes relate to the antigenic clusters as proposed by Smith et al. [18] or Du et al. [42]. A new antigenic variant differing by 2 or more epitopes from any previous cluster strain was grouped in a new cluster in agreement with Huang et al. [36]. This does not affect model dynamics, and relates only to the coloring of clusters in Figure 6 and Figure S4-A.

## Supporting Information

Accessions S1 **GenBank accession numbers used in reconstruction of** **figure 1** **phylogenetic tree.**
(CSV)

Figure S1 **Changes in prevalence for carrying strengths of strain competition and antigenic mutation rates.** Prevalence increases as crossimmunity between strains decreases, enabling multiple infections. When crossimmunity is 1, all strains are antigenically equal, and one lifetime infection is possible. When no crossimmunity is present, each antigenic-type can independently infect a host once.
(PDF)

Figure S2 **Changes in antigenic diversity and the McDonald-Kreitman related index (MKR) for varying strengths of strain competition and antigenic mutation rates.** **(A)** Mean Shannon diversity was measured for 40 years of simulation. Shannon diversity ranges from zero when a single circulating antigenic variant is present at each time point, to approximately 3.5nats when all the possible antigenic variants are continuously present. For a large range of the parameter space, stronger competition and lower mutation rates decrease Shanon diversity as fewer circulating antigenic types co-exist. **(B)** Single strain dominance based on the quantity ε from [22] (see Methods). With stronger competition and lower mutation rates epidemics are contain a larger fraction of a single antigenic type **(C)** Shannon diversity decreases with stronger positive selection (*ρ* = −0.88 when MKR>1) and with stronger negative selection (*ρ* = 0.39 when MKR<1). Positive selection roughly corresponds to lower mutation rates (ξ<10^−3^), while negative selection corresponds to higher mutation rates (ξ>10^−4^) **(D)** Single strain dominance increases with stronger positive selection (*ρ* = 0.88 when MKR>1) and an increases for stronger negative selection (*ρ* = −0.23 when MKR<1). (see methods for full description of epidemiological parameters).
(PDF)

Figure S3 **Seasonal patterns.** For simulations including metapopulation sinusoidal seasonal forcing was used (see Methods). Contact rate was modulated sinusoidally with 14% amplitude in temperate demes, and lower biannual seasonal cycles of weaker (7%) amplitude in the tropics. The observed seasonal patterns in the simulation include annual peaks centered around Jan–Feb in the northern hemisphere, July in the southern hemisphere and weaker peaks centered around late July and mid January in the tropics.
(PDF)

Figure S4 **Dynamic changes in the percentage of antigenic clusters and the dominance of antigenic variants in different metapopulation demes.** **(A)** Changes in the percentage of the population infected with a specific antigenic cluster variant for the northern hemisphere, the southern hemisphere and the tropics. On average we observe 13±6 antigenic clusters that come to dominate the virus population over the course of the 40 year simulation with an average duration of 4±2 years. One or two clusters usually dominate the deme population. Clusters are defined based on a threshold set when the cumulative change of two or more epitopes between any previous cluster antigenic-type is reached, based on [36] (see Methods). Clusters are only used for coloring of strains and figures and do not affect the model dynamics. A time window of 25 years was selected for comparison with [42] **(B)** Measurement of the onset time for all antigenic types (prior to cluster subdivision). Onset time was measured as the point where prevalence was estimated to reach 5% of its overall deme prevalence. Antigenic variants are more likely to reach significant prevalence in the tropics: 2±1.5 months earlier in the tropics compared to the northern hemisphere and 3±2 months earlier in the tropics compared to the southern hemisphere (p<0.001 for the combined results).
(PDF)

Figure S5 **Antigenic types across 40 years of simulated years.** A sample of antigenic types that emerge in the simulation are sampled and numbered sequentially. Red – Antigenic variants sampled from the trunk of the tree (fixed). Black – Antigenic variants sampled from sidebranches of the phylogenetic tree.
(PDF)

Figure S6 **Crossimmunity patterns for individual epitopes.** The thirteen most prevalent antigenic types from a span of 40 years of simulation were sampled (Figure 6) and ordered by year of introduction. Individual epitopes were compared between the strains. Epitopes with lower variability (2–3 variants per epitope) show a larger degree of reemergence while epitopes with higher variability (4–5 variants per epitope) show a lower degree of epitope reemergence.
(PDF)

Figure S7 **Full phylogenetic trees following the initial introduction of a virus with an increasing mutation rate.** Phylogenetic trees are based on samples of directly measured virus genealogy in the simulation (434 years). Figure 4 in the main body of the paper shows the last 40 years of a simulation with the same parameters (see caption of that figure for details). For these longer sampling windows, extinction was prevented by maintaining at least 50 infected individuals. **(A)** Model with no mutation. **(B)** Model with low mutation rate of ξ = 7.5×10^−6^. **(C)** Mutation rate of ξ = 7.5×10^−5^. **(D)** Mutation rate of ξ = 7.5×10^−4^. **(E)** Mutation rate of ξ = 7.5×10^−3^.
(PDF)

Table S1 **Model results for doubling the number of epitopes or variants per epitope.**
(PDF)
