# Supplementary figures and images for: The Roles of Competition and Mutation in Shaping Antigenic and Genetic Diversity in Influenza
Source: PLoS Pathog. 2013 Jan 3;9(1):e1003104. doi: 10.1371/journal.ppat.1003104 (PMC3536651; doi:10.1371/journal.ppat.1003104)

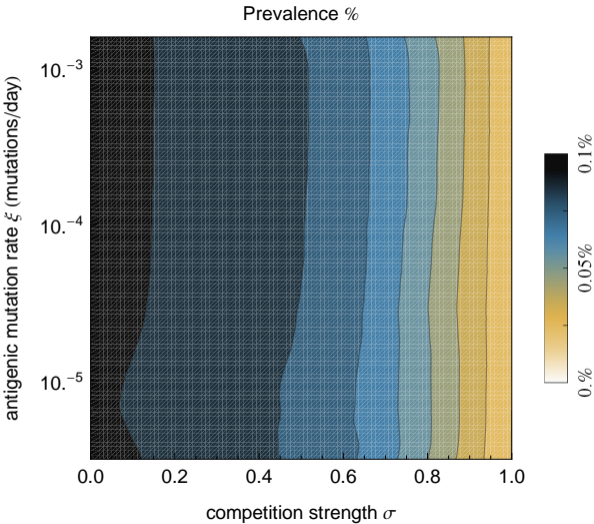

Supplement: Figure S1 — Changes in prevalence for carrying strengths of strain competition and antigenic mutation rates. Prevalence increases as crossimmunity between strains decreases, enabling multiple infections. When crossimmunity is 1, all strains are antigenically equal, and one lifetime infection is possible. When no crossimmunity is present, each antigenic-type can independently infect a host once. (PDF) [file ppat.1003104.s002.pdf]

**A**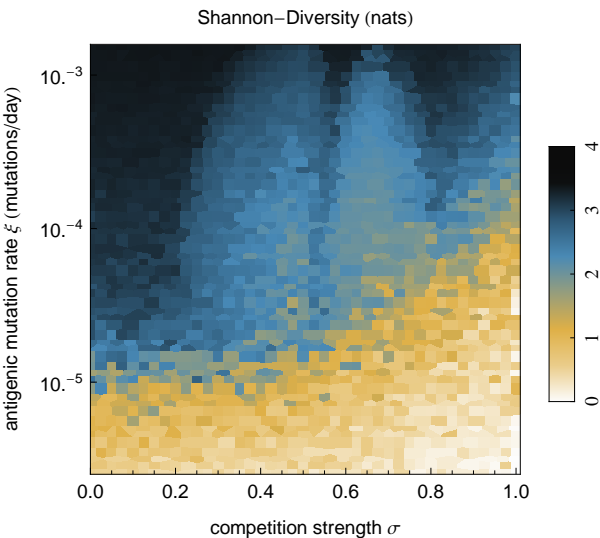**B**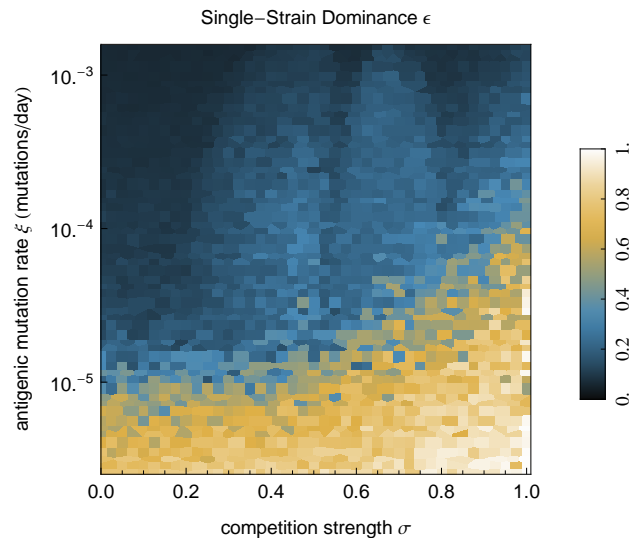**C**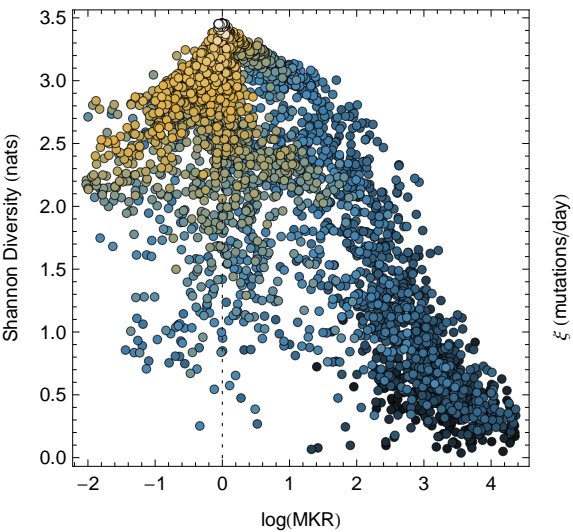**D**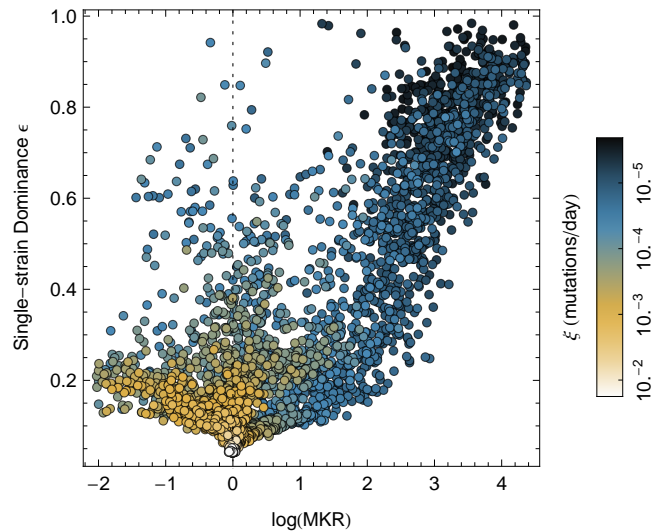

Supplement: Figure S2 — Changes in antigenic diversity and the McDonald-Kreitman related index (MKR) for varying strengths of strain competition and antigenic mutation rates. (A) Mean Shannon diversity was measured for 40 years of simulation. Shannon diversity ranges from zero when a single circulating antigenic variant is present at each time point, to approximately 3.5nats when all the possible antigenic variants are continuously present. For a large range of the parameter space, stronger competition and lower mutation rates decrease Shanon diversity as fewer circulating antigenic types co-exist. (B) Single strain dominance based on the quantity ε from [22] (see Methods). With stronger competition and lower mutation rates epidemics are contain a larger fraction of a single antigenic type (C) Shannon diversity decreases with stronger positive selection (ρ = −0.88 when MKR>1) and with stronger negative selection (ρ = 0.39 when MKR<1). Positive selection roughly corresponds to lower mutation rates (ξ<10−3), while negative selection corresponds to higher mutation rates (ξ>10−4) (D) Single strain dominance increases with stronger positive selection (ρ = 0.88 when MKR>1) and an increases for stronger negative selection (ρ = −0.23 when MKR<1). (see methods for full description of epidemiological parameters). (PDF) [file ppat.1003104.s003.pdf]

% infectious

North

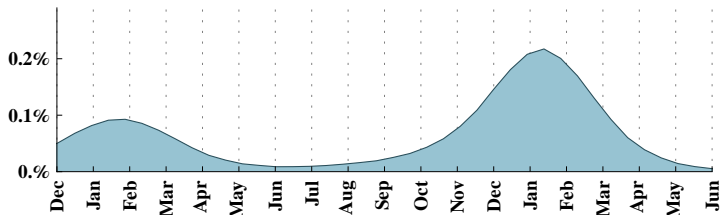

Tropics

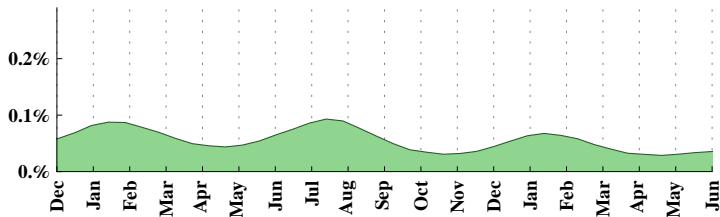

South

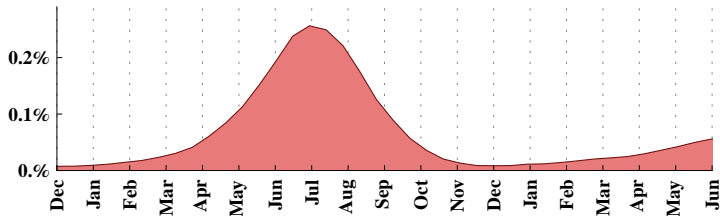

Supplement: Figure S3 — Seasonal patterns. For simulations including metapopulation sinusoidal seasonal forcing was used (see Methods). Contact rate was modulated sinusoidally with 14% amplitude in temperate demes, and lower biannual seasonal cycles of weaker (7%) amplitude in the tropics. The observed seasonal patterns in the simulation include annual peaks centered around Jan–Feb in the northern hemisphere, July in the southern hemisphere and weaker peaks centered around late July and mid January in the tropics. (PDF) [file ppat.1003104.s004.pdf]

A

% antigenic cluster

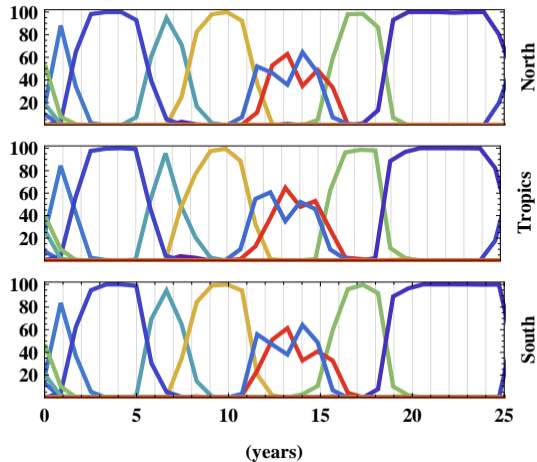

B

fraction

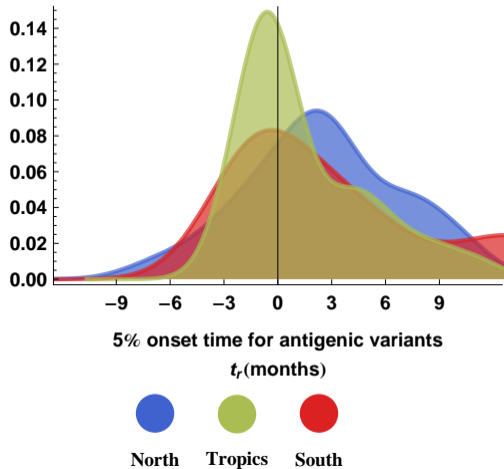

Supplement: Figure S4 — Dynamic changes in the percentage of antigenic clusters and the dominance of antigenic variants in different metapopulation demes. (A) Changes in the percentage of the population infected with a specific antigenic cluster variant for the northern hemisphere, the southern hemisphere and the tropics. On average we observe 13±6 antigenic clusters that come to dominate the virus population over the course of the 40 year simulation with an average duration of 4±2 years. One or two clusters usually dominate the deme population. Clusters are defined based on a threshold set when the cumulative change of two or more epitopes between any previous cluster antigenic-type is reached, based on [36] (see Methods). Clusters are only used for coloring of strains and figures and do not affect the model dynamics. A time window of 25 years was selected for comparison with [42] (B) Measurement of the onset time for all antigenic types (prior to cluster subdivision). Onset time was measured as the point where prevalence was estimated to reach 5% of its overall deme prevalence. Antigenic variants are more likely to reach significant prevalence in the tropics: 2±1.5 months earlier in the tropics compared to the northern hemisphere and 3±2 months earlier in the tropics compared to the southern hemisphere (p<0.001 for the combined results). (PDF) [file ppat.1003104.s005.pdf]

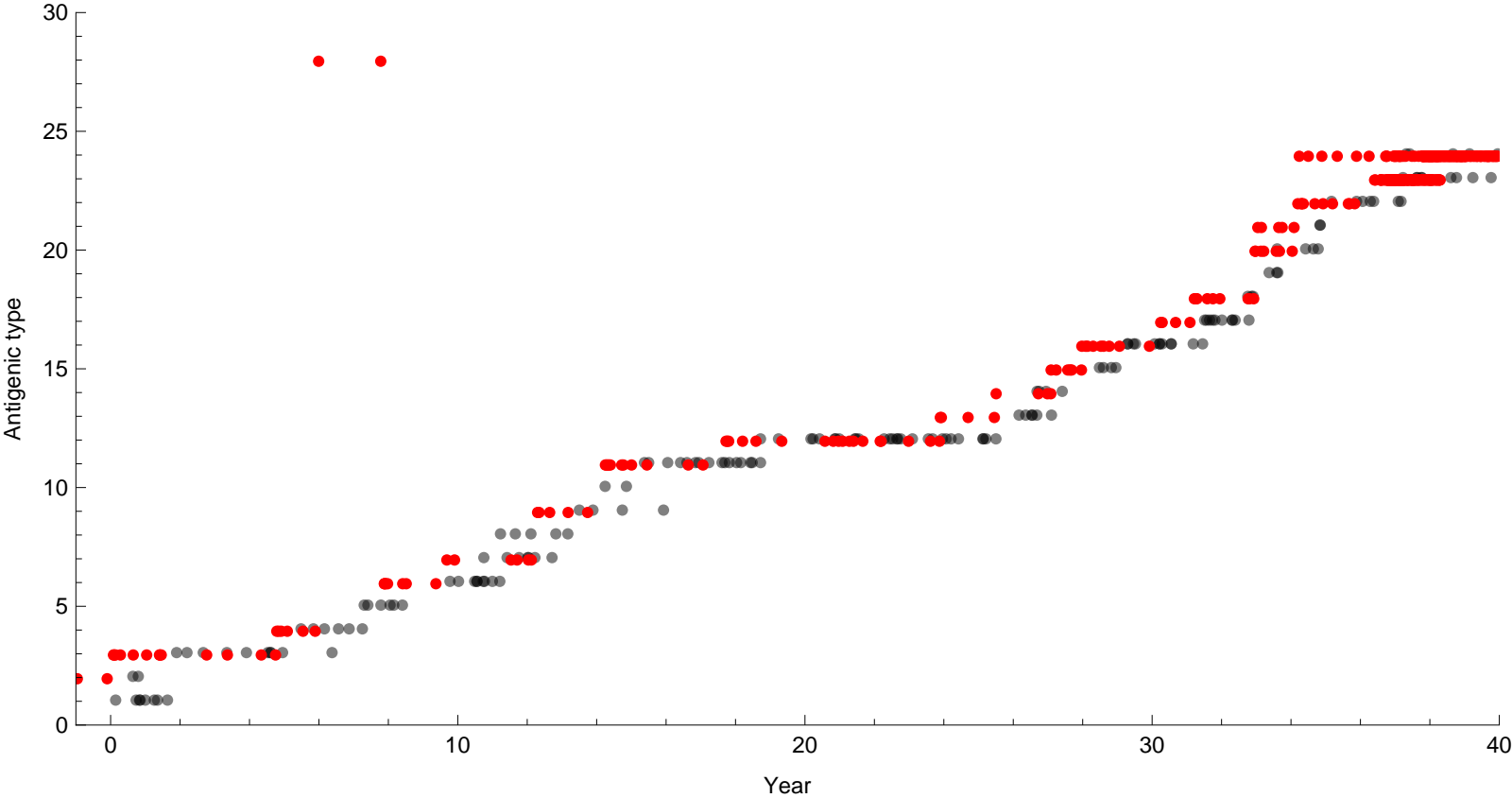

Supplement: Figure S5 — Antigenic types across 40 years of simulated years. A sample of antigenic types that emerge in the simulation are sampled and numbered sequentially. Red – Antigenic variants sampled from the trunk of the tree (fixed). Black – Antigenic variants sampled from sidebranches of the phylogenetic tree. (PDF) [file ppat.1003104.s006.pdf]

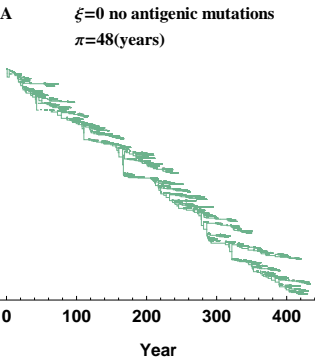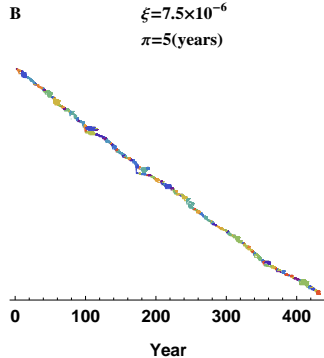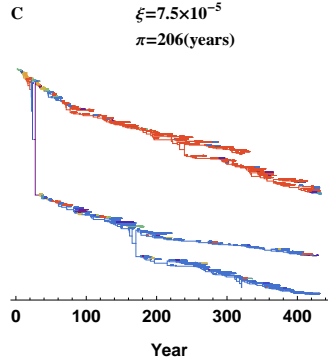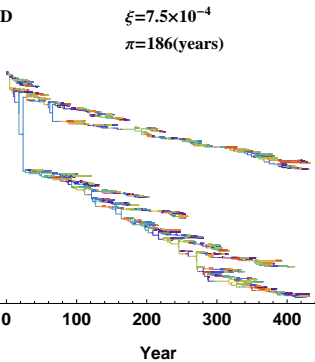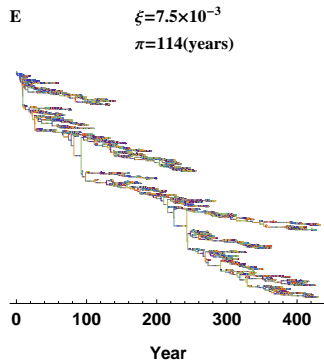

Supplement: Figure S7 — Full phylogenetic trees following the initial introduction of a virus with an increasing mutation rate. Phylogenetic trees are based on samples of directly measured virus genealogy in the simulation (434 years). Figure 4 in the main body of the paper shows the last 40 years of a simulation with the same parameters (see caption of that figure for details). For these longer sampling windows, extinction was prevented by maintaining at least 50 infected individuals. (A) Model with no mutation. (B) Model with low mutation rate of ξ = 7.5×10^−6^. (C) Mutation rate of ξ = 7.5×10^−5^. (D) Mutation rate of ξ = 7.5×10^−4^. (E) Mutation rate of ξ = 7.5×10^−3^. (PDF) [file ppat.1003104.s008.pdf]
