# Supplementary material for: The Roles of Competition and Mutation in Shaping Antigenic and Genetic Diversity in Influenza
Source: PLoS Pathog. 2013 Jan 3;9(1):e1003104. doi: 10.1371/journal.ppat.1003104 (PMC3536651; doi:10.1371/journal.ppat.1003104)
Supplement: Figure S6 — Crossimmunity patterns for individual epitopes. The thirteen most prevalent antigenic types from a span of 40 years of simulation were sampled (Figure 6) and ordered by year of introduction. Individual epitopes were compared between the strains. Epitopes with lower variability (2–3 variants per epitope) show a larger degree of reemergence while epitopes with higher variability (4–5 variants per epitope) show a lower degree of epitope reemergence. (PDF) [file ppat.1003104.s007.pdf]

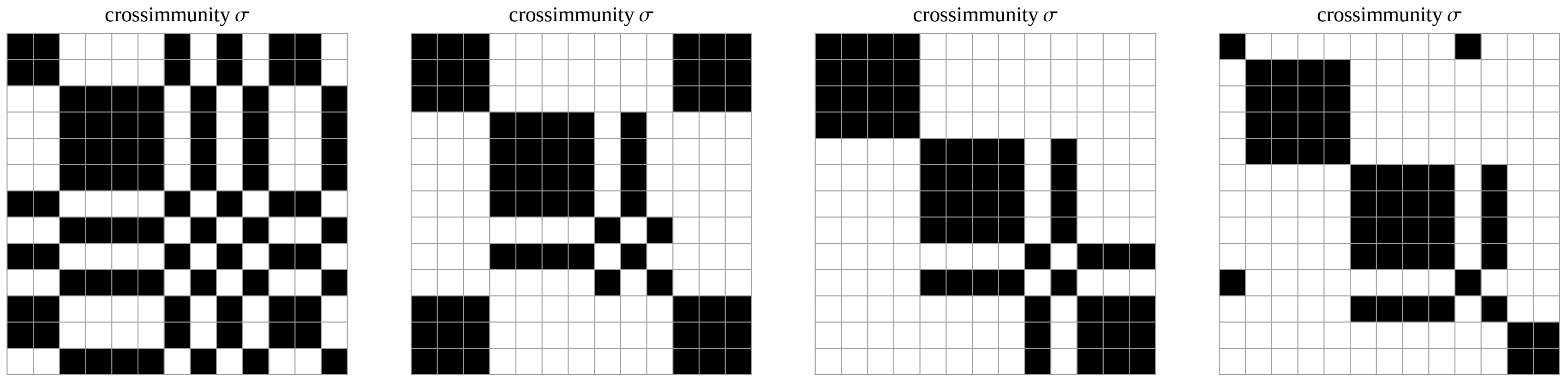

Epitope with 2 variants Epitope with 3 variants Epitope with 4 variants Epitope with 5 variants
