# Supplementary material for: The Roles of Competition and Mutation in Shaping Antigenic and Genetic Diversity in Influenza
Source: PLoS Pathog. 2013 Jan 3;9(1):e1003104. doi: 10.1371/journal.ppat.1003104 (PMC3536651; doi:10.1371/journal.ppat.1003104)
Supplement: Table S1 — Model results for doubling the number of epitopes or variants per epitope. (PDF) [file ppat.1003104.s009.pdf]

Table S1. Model results for doubling the number of epitopes or variants per epitope

Epitope Configuration	Total Mutation Rate Across Epitopes (1/day)	Immunity Reduction Per Epitope Change (1-σ)	Mean Genetic Diversity π	Incidence Increase / Decrease
5x4x3x2	8×10^{-6}	13%	5.7±0.1	Baseline
5x5x4x4x3x3x2x2	8×10^{-6}	13%	10±4	↑
5x5x4x4x3x3x2x2	8×10^{-6}	6.5%	6±1	≈
5x5x4x4x3x3x2x2	1.6×10^{-5}	6.5%	8±5	≈
10x8x6x4	8×10^{-6}	13%	16±15	↑
10x8x6x4	8×10^{-6}	6.5%	6±1	↓
10x8x6x4	1.6×10^{-5}	6.5%	10±5	↓
